# Correlative Multi-scale Cryo-imaging Unveils SARS-CoV-2 Assembly and Egress

**DOI:** 10.21203/rs.3.rs-134794/v1

**Published:** 2021-01-19

**Authors:** Peijun Zhang, Luiza Mendonca, Andrew Howe, James Gilchrist, Dapeng Sun, Michael Knight, Laura Zanetti-Domingues, Benji Bateman, Anna-Sophia Krebs, Long Chen, Julika Radecke, Yuewen Sheng, Vivian Li, Tao Ni, Ilias Kounatidis, Mohamed Koronfel, Marta Szynkiewicz, Maria Harkiolaki, Marisa Martin-Fernandez, William James

**Affiliations:** University of Oxford; University of Oxford; Diamond Light Source; Diamond Light Source; University of Pittsburgh; University of Oxford; Science & Technology Facilities Council; Research Complex at Harwell; University of Oxford; University of Oxford; Diamond Light Source; Diamond Light Source; University of Cambridge; University of Oxford; Diamond Light Source; Diamond Light Source; Central Laser Facility; Diamond Light Source; Rutherford Appleton Laboratory; University of Oxford

**Keywords:** SARS-CoV-2, COVID-19, cryoEM, cryoET, subtomogram averaging, cryoFIB/SEM, soft X-ray, cryo-tomography, virus assembly, egress, spike

## Abstract

Since the outbreak of the SARS-CoV-2 pandemic, there have been intense structural studies on purified recombinant viral components and inactivated viruses. However, structural and ultrastructural evidence on how the SARS-CoV-2 infection progresses in the frozen-hydrated native cellular context is scarce, and there is a lack of comprehensive knowledge on the SARS-CoV-2 replicative cycle. To correlate the cytopathic events induced by SARS-CoV-2 with virus replication process under the frozen-hydrated condition, here we established a unique multi-modal, multi-scale cryo-correlative platform to image SARS-CoV-2 infection in Vero cells. This platform combines serial cryoFIB/SEM volume imaging and soft X-ray cryo-tomography with cell lamellae-based cryo-electron tomography (cryoET) and subtomogram averaging. The results place critical SARS-CoV-2 structural events – e.g. viral RNA transport portals on double membrane vesicles, virus assembly and budding intermediates, virus egress pathways, and native virus spike structures from intracellular assembled and extracellular released virus - in the context of whole-cell images. The latter revealed numerous heterogeneous cytoplasmic vesicles, the formation of membrane tunnels through which viruses exit, and the drastic cytoplasm invasion into the nucleus. This integrated approach allows a holistic view of SARS-CoV-2 infection, from the whole cell to individual molecules.

## Introduction

Since December 2019, the world has been in the middle of what has been dubbed the “greatest pandemic of the century”. The etiological agent was named Severe Acute Respiratory Syndrome Coronavirus 2 (SARS-CoV-2) and the disease caused by it Coronavirus Disease 2019 (COVID-19) ^1–3^. Coronaviruses are small enveloped viruses with positive non-segmented RNA genome. Among RNA viruses, Coronaviruses bear one of the largest genomes and its replication in the cell is complex involving frameshift slipping and replicase jumps with abundant RNA duplexes being generated. Coronaviruses, like most RNA viruses, induce the development of a range of membrane compartments that seclude and protect the viral components contributing to increased replication eficiency and innate immune recognition escape ^4–9^.

All coronavirus structural proteins arise from the translation of positive-sense subgenomic RNA, which in turn are generated by replicase jumps when the negative strand copy of the viral genome is replicated. The S protein makes the viral spike, responsible for cellular attachment, entry, and fusion. It adopts two main conformations: prefusion, composed of trimers of S1 and S2, and postfusion, a non-active conformation composed solely of S2 ^10–17^. The N protein is responsible for encapsidating and protecting the genomic viral RNA, forming ribonucleoprotein (RNP) complexes that reside in the internal space of the viral particle. The E protein is the smallest of the structural proteins and is thought to act as an ion channel ^18^. The M protein is the most abundant protein in SARS-CoV-2 and is a transmembrane protein that lines the internal surface of the virus lipid membrane ^19^.

SARS-CoV-2 cycle starts with S interaction with ACE2 in the host cell surface ^12–14,16,17,20^. This interaction can either be followed by S2’ cleavage at the cell surface by TMPRSS2, or trigger the endocytosis of the viral particle, when TMPRSS2 is not present ^12^. Upon a second still not completely characterized trigger, which may be the S2’ site cleavage (by TMPRSS2 or endosomal proteases) and/or endosomal acidification, the spike changes conformation and inserts its fusogenic peptide into the host membrane to fuse it with the viral envelope, after which the spike finally adopts the postfusion conformation ^10,11,21,22^. The viral contents are then released into the cytoplasm, and the precursor polyproteins Pp1a and Pp1ab are synthetized. Non-structural proteins 3, 4 and 6, which are part of Pp1a/Pp1ab, induce the formation of secluded, often interconnected, membranous compartments known as DMVs (Double Membrane Vesicles) ^23–25^. The DMVs compartmentalize the Replication Transcription Complexes (RTCs) and are the sole compartments where viral genome replication takes place ^6^, both for the synthesis of the negative strand viral RNA and for the synthesis of the positive strand viral genome RNA and subgenomic mRNAs. Initially, it was thought that these compartments were sealed and had no connection to the cytoplasm, raising the mystery of how the mRNAs could reach the cytoplasm to be translated by the cellular ribosomes. Recently, however, a molecular pore has been described in MHV and SARS-CoV-2 that can serve as export portal for the mRNA and positive strand viral genome copies ^26^. The assembly of the viral particle is thought to take place at modified cellular membranes derived from the ER, Golgi and ERGIC (endoplasmic-reticulum-Golgi intermediate compartment), and viral release through exocytosis based on studies of other coronaviruses ^27–30^. A recent fluorescence microscopy study suggests SARS-CoV-2 release through lysosomal exocytosis ^31^. Although, there have been intense structural studies on recombinant viral components and purified inactivated viruses ^12–14,16,17,20,32–34^, structural investigation of the SARS-CoV-2 replication process in the native cellular context is scarce ^35^, and viral assembly and egress are still not well understood.

In this study we exploited a unique correlative multi-modal multi-scale cryo-imaging approach to investigate SARS-CoV-2 replication in Vero cells under near-native conditions. This approach empowers a holistic view of SARS-CoV-2 infection, from the whole cell to individual virus spike molecules, revealing new pathways of SARS-CoV-2 assembly and egress and cytopathic effects of SARS-CoV-2 infection.

## Results

### SARS-CoV-2 replication induces profound cytopathic effects in host cells

To image and investigate SARS-CoV-2 replication in near-native cell context, we infected Vero cells grown on indexed EM grids with SARS-CoV-2 at 0, 0.1 and 0.5 multiplicity of infection (MOI). At 24 hours post infection (hpi), the cells were fixed with 4% paraformaldehyde and plunge frozen in liquid ethane. As illustrated in the workflow (Figure S1), cryoEM grids containing SARS-CoV-2 infected cells were first imaged in a Titan Krios to identify each individual infected cell (39.2 % for MOI of 0.1 and 45.4% for MOI 0.5) where cryoET tilt series were collected first at the cell periphery. The grids were then transferred to a FIB/SEM dualbeam instrument and the exact same infected cells were imaged with serial cryoFIB/SEM volume imaging ^36^ or cryoFIB milling of cellular lamellae at the target region where additional cryoET tilt series were collected ^37^. Alternatively, we imaged the same infected cells on cryoEM grids by soft X-ray cryo-tomography ^38^. These imaging modalities provide the necessary structural and ultrastructural information at different length scales to visualise the infecting viruses in their cellular context and are highly complementary. Indeed, such a unique approach enabled the direct visualisation of the SARS-CoV-2 replication and cytopathic effects in a multi-modal, multi-scale and correlative manner.

**Figure 1:**
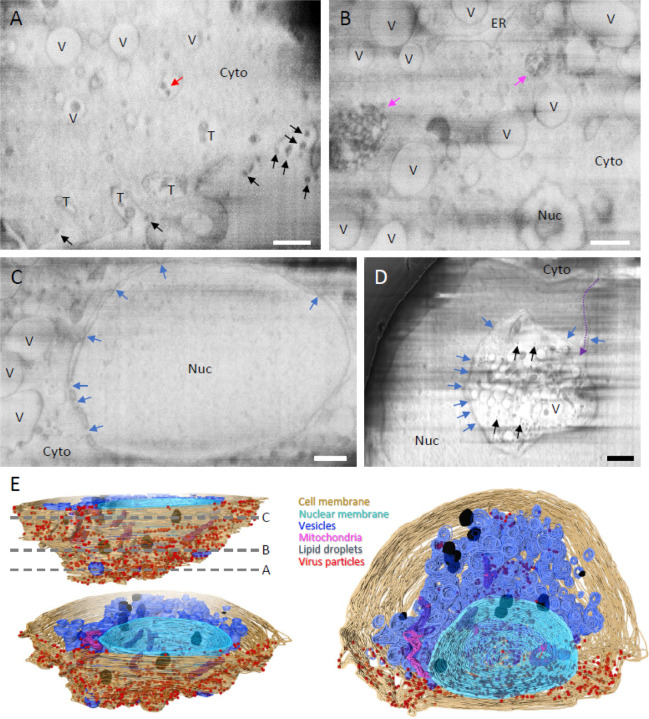
Serial cryoFIB/SEM volume imaging of entire SARS-CoV-2 infected cell. (A-D) Representative cryoFIB/SEM slices of a SARS-CoV-2 infected cell at the cell periphery (A), cytoplasm (B), cell nucleus (C), and invagination of cytoplasm into the nuclear space (note, from a different cell) (D). Scale bars, 500 nm in A-C, 1 µm in D. Black and red arrows, extracellular and intracellular virus particles; blue arrows, nuclear pores; pink arrows, complex membrane compartment; dashed purple arrow, invagination path; V, vesicles; T, tunnels; Nuc, nucleus; Cyto, cytoplasm; ER, Endoplasmic reticulum. (E) Surface rendering of the segmented volume of SARS-CoV-2 infected cell shown in A-C. Segmented organelles and virus particles are labeled with colors indicated. The dashed lines (E, top left panel) indicate the positions of slices shown in A-C, respectively.

Compared to uninfected cells (Figure S2, Movie 1), serial cryoFIB/SEM images of SARS-CoV-2 infected cells display an extensive array of cytopathological alterations throughout the entire cell, as illustrated in Figure 1 and Movies 2–5. At the cell surface, there were many virus-containing membrane tunnels extending deep into the cell (Figure 1A, “T”, Movie 2), resembling those in HIV-1 infected microphages ^39^. CryoET confirms the presence of SARS-CoV-2 particles just outside of cells and in membrane tunnels. In addition, virus particles were also found within intracellular membrane vesicles that are not connected to cell membrane (Figure 1A, red arrow). Deep into the cell, we found that much of the cytoplasm (Fig. 1B), especially the paranuclear region (Fig. 1C), is occupied with abundant membrane compartments of different morphologies, including numerous vesicles (“V”), the endoplasmic reticulum (ER) and the nucleus (“Nuc”) (Movies 2–4). CryoET of cell lamellae containing these vesicles confirmed that they are the so-called “double membrane vesicles” (DMVs) (Fig. 2A and B) where viral genome replication takes place ^6^. Nuclear pores are clearly distinguishable in both SARS-CoV-2 infected and uninfected cells (Figure 1C, Figure S2A and C, blue arrows). We frequently found electron-dense complex membrane compartments in infected cells (Figure 1B, pink arrows). A more striking feature observed in infected cells is the cytopathic damage to the nucleus compared to the control cells (Figure 1D, Figure S3G), where, in extreme cases, nearly a half of the nucleus has been taken up by the invaginated cytoplasm (Movie 5). Such cytoplasm invagination was also noticed in a conventional EM image of stained plastic sections of SARS-CoV-2 infected cells ^40^.

Exploiting high throughput whole-cell imaging capability offered by soft X-ray cryo-tomography, we analysed many targeted infected and uninfected cells that had been identified and imaged by cryoEM/ET at the cell periphery. At the whole cell level, soft X-ray images show substantial mitochondria morphological changes. We observed that long tubular shaped mitochondria in the uninfected cells (Figure S3A, C, yellow arrows) have been mostly disrupted in the infected cells (Figure S3B, D). Consistently, we observe numerous vesicles at perinuclear regions (Figure S3F) and cytoplasmic invaginations (Figure S3G) in the infected cells.

### SARS-CoV-2 RNA synthesis and transport

The first step in SARS-CoV-2 production is viral genome replication. Coronaviruses have evolved a sophisticated RNA replication strategy for the generation of the genomic negative-sense and subgenomic RNAs, which relies heavily on double stranded RNA intermediaries, a potent activator of RIG-I and MDA-5 ^41–43^. Thus, cellular compartmentalization of RNA transcripts serves as an innate immune evasion strategy. DMVs are induced during the replication of a variety of RNA viruses ^4,5,7–9^ and were identified as the sole compartment where viral RNA transcription occurs for coronaviruses ^6^. Indeed, cryoET of cell lamella revealed that abundant intracellular vesicles observed in the 3D volume of infected cell (Figure 1B, Movies 2–4) are DMVs likely containing viral RNA transcripts as previously suggested 6,44 (Figure 2A-C, Movie 6). There are also a substantial amount of vesicle packets (VPs) (Figure 2A) ^29^, apparently resulting from the fusion of the outer membranes of DMVs. Since the sample was cryofixed 24 hours post infection, this is consistent with a previous observation that the number of VPs increases with the time of infection ^6^. Until very recently, DMVs were thought to be completely enclosed, which raised the question of how the viral mRNAs could gain access to the cytoplasm to be translated. We observed several double-membrane-spanning pore complexes in DMVs (Figure 2B-D, yellow arrow), resembling the RNA transport portal observed in DMVs of murine hepatitis coronavirus (MHV) infected cells in a recent study ^26^. However, the portal appears rare in DMVs of SARS-CoV-2 (total 9 portals from 24 DMVs) compared to those of MHV (average 8 portals per DMV, or 192 for 24 DMVs) (Wolff et al., 2020a), signifying a difference between coronaviruses.

### SARS-CoV-2 assembly and budding

**Figure 3:**
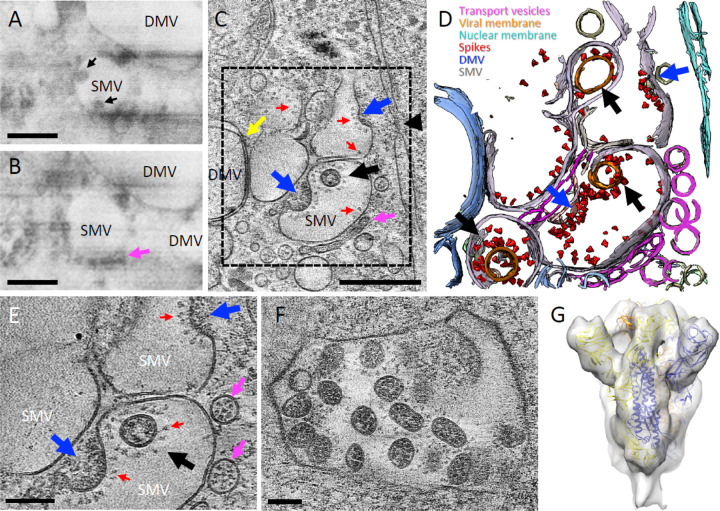
SARS-CoV-2 cytoplasmic viral assembly. (A-B) CryoFIB/SEM images of two sequential slices separated by 80 nm. Black arrows point to virus particles in single membrane vesicle (SMV). Pink arrow points to small dense vesicles lining the outside of virus-containing SMV. (C) Tomographic slice of cryoFIB lamella depicting SARS-CoV-2 assembly, with DMV portals (yellow arrow), assembling viruses (blue arrow), assembled virus (black arrow), viral spikes on SMV membranes (red arrows), dense vesicles around the assembly site (pink arrow, as in B) and a nucleopore (black arrowhead). (D) Density segmentation of C, displaying three virus particles (black arrows) and two assembly sites (blue arrows). (E) An enlarged view (at a different angle) of boxed area in C, showing assembled virus (black arrow), assembling viruses (blue arrows), spikes (red arrows) and spike-containing vesicles (pink arrows). (F) Large intracellular virus-containing vesicle (LVCV) full of readily assembled viruses. (G) Subtomogram average of viral spikes of intracellular viruses from cell lamellae at 11 Å resolution, fitted with an atomic model of spike trimer (PDB 6ZB5) 48. Scale bar is 300 nm in A, B and C; and 100 nm in E and F.

The translation of the subgenomic vRNAs gives rise, amongst others, to the structural proteins N, M, E and S, which are required for assembly. M, E and S are membrane-associated proteins and are localized to the ER, Golgi and the ERGIC ^30,44^. The N protein associates with the genomic vRNA and M protein, which presumably drives vRNA packaging and genome encapsidation ^45,46^. The main assembly and budding site of other coronaviruses has been previously described at the ERGIC by conventional EM of stained plastic sections ^7,28,30,44^. In serial cryoFIB/SEM images of SARS-CoV-2 infected cells, we observed vesicles containing virus particles (Figure 3A, black arrows), along with a string of small dense vesicles (Figure 3B, pink arrow) lining along the vesicle membrane. The same architecture was captured by high-resolution cryoET of cell lamella from a similar perinuclear region, which shows these are in fact SARS-CoV-2 assembly and budding sites (Figure 3C–E). CryoET and subtomogram averaging further revealed that the small dense vesicles are SARS-CoV-2 spike containing transport vesicles (Figure S4), possibly supplying newly synthesized spikes and other viral components via fusion with the single membrane vesicles (SMVs) where viral assembly takes place (Figure 3C–E pink arrows, Movie 7). Indeed, spikes are observed on SMV membranes sparsely distributed or otherwise clustered at the assembly sites (Figure 2C, Figure 3C–E, red arrows, Movie 7). Interestingly, several SARS-CoV-2 assembly intermediates were observed within a single tomogram from a cell lamella (Figure 3C–E, blue arrows, Movie 7), along with fully assembled virus particles released into SMVs (Figure 3C–E, black arrows, Movie 7), thus capturing the assembly and budding process of SARS-CoV-2. It is conceivable that upon fusion of transport vesicles with the SMV, spikes are readily diffused on the SMV membrane. They cluster when interacting with N-associated vRNA, possibly via M protein ^19,45^, which initiates the assembly and budding process that finally releases the viral particle into the SMV. Consistent with this, spike clusters are observed exclusively associated with the agglutination/gathering of electron dense material, which presumably represents viral genome. Noticeably, the virus assembly site is frequently present in the vicinity of RNA portals in DMVs (Figure 2B-D, yellow arrows), potentially facilitating the assembly process.

Most virus particles are found in SMVs, some contain a single virion, while others encompass multiple virions (Figure 3E–F). CryoET and subtomogram averaging of 450 spikes from these particles yielded a density map at 11 Å resolution (at 0.143 FSC cut-off) by emClarity ^47^ (Figure 3G, Figure S5). The averaged density map resolves the overall spike structure, which overlaps very well with prefusion spike atomic models ^12–17^ (Figure 3G). Some virus particles were also observed in electron-dense complex membrane compartments (CMC) (Figure 4). There were two types of virus particles: Viruses protected by single membrane vesicles (SMV) in CMC show prefusion spikes (Figure 4D, G-H); viruses in the lumen of CMC, however, have either no spikes (Figure 4B) or a few postfusion spikes on their surfaces (Figure 4E-F). These virus particles could be off-pathway viral assemblies (in the case of SMV-protected viruses displaying prefusion spikes), or remnants of late endosomes from viral entry or lysosomes for viral degradation. The fact that the spike proteins are in the postfusion state suggests that proteolytic processing has taken place in these compartments resulting in S1 shedding. Therefore, we suggest that assembly at the SMVs is the only pathway which will lead to infectious viral progeny.

### SARS-CoV-2 egress

There has not been much detailed studies on how SARS-CoV-2 viruses are released from cell. We investigated SARS-CoV-2 egress using both serial cryoFIB/SEM volume imaging and cryoET. CryoFIB/SEM images reveal abundant virus exiting tunnels in 3D at the cell periphery connecting to cell membrane (Figure 5A-B, Movie 2). This likely resulted from the fusion of very large multi-virus containing vesicles with cell membrane, i.e. egress through exocytosis-like mechanism. Consistent with cryoFIB/SEM analysis, we also observed virus exiting tunnels in cryo-tomograms (Figure 5C). The fact that these compartments often contained many viral particles suggests that this is a snapshot of viral exit, rather than cellular entry.

In addition to exiting through tunnels, we also frequently found plasma membrane discontinuities, or membrane lesions, next to virus particles outside the cell (Figure 5E). The membrane lesions are mostly discrete in appearance, which argues against them being an artifact of sample preparation. There were 116 membrane lesion sites in 74 tomograms, and 44.6% of tomograms show cell membrane lesions in infected cells (Figure 5D-E, Movies 8–9), whereas 18.7 % tomograms from uninfected cells display similar but more sparse membrane lesions (10 membrane lesion sites from 16 tomograms). Close inspection of individual membrane lesions indicates that the underlying cytoskeleton, such as actin filaments, is largely retained (Figure 5E inset). The fact that we observed similar membrane lesions, but to a lesser extent in control cells, suggests that SARS-CoV-2 may exploit the host cell machinery for its egress. It is unclear whether the cell can recover from such membrane wounds, or if exit through membrane lesion is a sign of late infection and will eventually lead to cell lysis and death.

CryoET subtomogram averaging of 7090 spikes from extracellular virus particles yielded a density map at 8.7 Å resolution (at 0.143 FSC cut-off), which represents the prefusion state (Figure 5F, Figure S5). Spike structures from intracellular and extracellular viruses agree with each other very well (Figure 5G), suggesting that no further substantial structure rearrangement takes place for viral spikes from assembly to egress, given the current resolution. While all previous spike structures are either from recombinant proteins or from purified inactivated virus particles ^10–17,32–34^, the two spike structures presented here were derived directly from infected cells in the cellular context, and thus represent the closest to the native condition.

## Discussion

We used a correlative approach to image the SARS-CoV-2 infection in near-native frozen cells, encompassing multiple spatial scales, from the whole cell level to the subcellular and molecular levels, thereby providing the first, to our knowledge, holistic view of the infection process. Crucially, the novel integration of multi-scale imaging data achieved through this workflow (Figure S1), has allowed us to directly visualise the structural events of SARS-CoV-2 replication in the context of the cytopathic consequences to the infected cell. Given prior knowledge of SARS-CoV-2 and other coronaviruses from previous studies ^6,8,28^, these images led us to propose a model for SARS-CoV-2 replication, in particular virus genome replication, assembly and egress. Images of the replication process of SARS-CoV-2 reveal a spatially well-organized and highly efficient process. From genome replication, to protein synthesis and transport, to virus assembly and budding, these processes take place in close-knit cytoplasmic compartments. As illustrated in Figure 6, RNA replication, including genomic vRNA and subgenomic mRNA, occurs in DMVs, secluding them from innate immune response (step 1). The newly synthesized vRNAs are then transported out of DMVs through the transmembrane portals to virus assembly sites proximal to DMVs and portals (step 2a), whereas mRNAs exit through the same portal to cytoplasm and ER/Golgi for protein production (step 2b). The viral spikes, in a trimeric prefusion form produced and matured in ER/Golgi network, are transported to the assembly sites via small transport vesicles (step 3). Upon fusion of transport vesicles with SMV membranes, viral spikes cluster at the assembly site where vRNA and N protein are present, resulting in a positive membrane curvature and finally bud into the SMV (step 4). The assembly and budding process give rise to large virus containing vesicles (LVCV) which contain multiple virus particles and small (or single) virus-containing vesicles (SVCV). Virus particles in LVCVs could exit through tunnels via exocytosis, perhaps by lysosomal exocytosis ^31^ (step 5a). Viruses in the single virus-containing vesicle (SVCV) may exit via cell membrane lesions (step 5b), although it is not clear what is the mechanism exploited by the virus. Many aspects of this model are still speculative and require additional experimental validation and testing it with relevant human cells, such as lung cells.

A holistic understanding of the genome replication, assembly and egress of the SARS-CoV-2 virus during its journey through the cell is critically important as it bears the means to inspire new medical interventions to stop infection. There are many facets of this process awaiting further investigation to dissect the molecular mechanism of SARS-CoV-2 replication, including the roles of other viral proteins, such as M and E, as well as host proteins and machines. Nevertheless, this study provides a first direct look of the SARS-CoV-2 replication cycle under near-native conditions and structures of prefusion spikes directly from cellular assembled and extracellular released virus particles. We hope the data presented here will inspire much additional work. The unique methodologies and workflow developed through this study can be broadly applied to studies of infection processes of other human pathogens beyond SARS-CoV-2.

## Methods

### Cell lines and viruses

African green monkey kidney Vero Ccl-81 cells Female (ATCC, CCL-81) were maintained in Dulbecco Modified Eagle media supplemented with 5% Fetal Bovine Serum, 10 units/mL penicillin (Gibco), 10 µg/mL streptomycin (Gibco), and 2mM l-glutamine. Cell line has not been authenticated.

SARS-CoV-2 isolate BetaCoV/England/02/2020 (EPI_ISL_407073) was deposited by Professor Maria Zambon and obtained through BEI Resources, NIAID, NIH: SARS-Related Coronavirus 2, Isolate England/02/2020, NR-52359. The viral stock used in this study has been sequenced to confirm retention of the furin cleavage site through the amplifying passages.

### Sample preparation

Vero Ccl-81 cells (ATCC) were maintained in Dulbecco Modified Eagle media supplemented with 5% Fetal Bovine Serum 10 units/mL penicillin (Gibco), 10 µg/mL streptomycin (Gibco), and 2mM l-glutamine (Gibco). 16,000 cells were seeded on the carbon-side of fibronectin treated G300F1 R2/2 gold EM grids in a 6-well plate well. Infections were performed using passage 3 of SARS-CoV-2 England/02/2020 at MOI of 0.5, 0.1 or 0 (for negative controls). Media was removed from the Vero Ccl-81 cells (ATCC) and replaced with an appropriate amount of virus diluted in 0.5 mL of Dulbecco’s modified Eagle medium (Merck) with 1% FCS, 10 units/mL penicillin (Gibco), 10 µg/mL streptomycin (Gibco), and 2mM l-glutamine (Gibco). The cells were incubated at room temperature for 15 minutes after which a further 1.5 mL of media was added to each well. The plate was then incubated at 37°C for 24 hours following which supernatants were discarded and cells washed with 2 mL of PBS. The cells were then fixed by addition of 3 mL of 4% paraformaldehyde in PBS for 1 hour at room temperature. After fixation, grids were plunge-frozen on a Leica grid plunger GP2. 1 µl of concentrated 10 nm gold fiducials was applied to the gold-side of the EM grid and blotted from the gold-side. The grid was quickly immersed in liquid ethane after blotting. Frozen grids were stored in liquid nitrogen until data collection.

### CryoET data acquisition

Tilt series acquisition was carried out at a FEI Titan Krios G2 (Thermo Fisher Scientific) electron microscope operated at 300 kV and equipped with a Gatan BioQuantum energy filter and post-GIF K3 detector (Gatan, Pleasanton, CA).

Tilt series were recorded using SerialEM tilt series controller with pixel sizes of 1.63 Å, 2.13 Å and 4.58 Å for intact cells and 2.13 Å and 7.58 Å on lamella. Zero-loss imaging was used for all tilt series with a 20 eV slit width. Defocus values ranged from −2 µm to −7 µm, except for lamella at 7.58 Å pixel size where 50 µm defocus was used. A 100 µm objective aperture was inserted. A grouped dose-symmetric scheme was used for all tilt-series; intact cells were collected with a range of +/−60 degrees at 3 degree increments in groups of 3 and total dose of 120–135 e/Å^2^; lamella with +/−54 degrees at 3 degree increments and groups of 3 with total dose of 120–135 e/Å^2^ at 4.58 Å and +/−54 degrees at 3 degree increments and groups of 10 with total dose of 70–90 e/ Å^2^ at 7.58 Å. Autofocus and tracking was performed at each tilt with drift measurement taken at tilt reversals with a 10 Å/s target rate. At each tilt, 5 movie frames were recorded using Correlated Double Sampling (CDS) in super-resolution mode and saved in lzw compressed tif format with no gain normalisation. Movies were subsequently gain normalised during motion correction and Fourier cropped back to physical pixel size. After each tilt-series a script was run to take a fresh dark reference and reset the defocus offset. A total of 294 tilt series were collected, of which 56 tilt series from control uninfected cells (20 from cell lamella and 36 from cell periphery) and 238 tilt series from SARS-CoV-2 infected cells (90 from lamella and 148 from cell periphery).

### CryoFIB lamella preparation

Lamella milling of SARS-Cov-2 infected cells was carried out using a Scios DualBeam cryoFIB (ThermoFisher Scientific) equipped with a PP3010T transfer system and stage (Quorum Technologies). Grids were sputter coated within the PP3010T transfer chamber maintained at −175 °C. After loading onto the Scios stage at −168 °C, the grids were inspected using the SEM (operated at 5 kV and 13 pA) and infected cells identified by correlation from TEM. The grid surface was coated using the gas injection system (Trimethyl(methylcyclopentadienyl)platinum(IV), ThermoFisher Scientific) for 3 s, yielding a thickness of ~3 µm. Milling was performed using the ion beam operated at 30 kV and currents decreasing from 300 pA to 30 pA. At 30 pA lamella thickness was less than 300 nm. During the final stage of milling, SEM inspection of the lamellae was conducted at 2 kV and 13 pA.

### Serial cryoFIB/SEM volume imaging

Samples were imaged on a Zeiss Crossbeam 550XL fitted with a Quorum transfer station and cryo-stage. They were mounted on a Quorum-compatible custom sample holder and coated with platinum for 60 sec at 10 mA on the Quorum transfer stage, prior to loading on the cryo-stage. Stage temperature was set at −165°C, while the anticontaminator was held at −185°C.

Samples were imaged at 45° tilt after being coated again with Pt for 2× 30sec using the FIB-SEM’s internal GIS system, with the Pt reservoir held at 25°C. Initial trapezoid trenches were milled at 30kV 7 nA over 15 μm to reach a final depth of 10 μm, with a polish step over a rectangular box with a depth of 10 μm performed at 30kV 1.5 nA. Serial Sectioning and Imaging acquisition was performed as follows: FIB milling was set up using the 30kV 700 pA probe, a z-slice step of 20 nm and a depth of 10 μm over the entire milling box; SEM imaging was performed at a pixel depth of 3024×2304 pixels, which resulted in a pixel size of 6.5 nm, with the beam set at 2kV 35pA, dwell time 100 nsec and scan speed 1, averaging the signal over 100 line scans as a noise-reduction strategy.

### CryoET image processing

The frames in each tilt angle in a tilt series were processed to correct drift using MotionCor2 ^49^. For the intact cells dataset, all tilt series were aligned using the default parameters in IMOD version 4.10.22 with the eTomo interface, using gold-fiducial markers ^50^. For lamella dataset, tilt series were aligned in the framework of Appion-Protomo fiducial-less tilt-series alignment suite ^51^. After tilt series alignment, the tilt-series stacks together with the files describing the projection transformation and fitted tilt angles were transferred to emClarity for the subsequent subtomogram averaging analysis ^47^.

### Subtomogram averaging

All subtomogram averaging analysis steps were performed using emClarity, mostly following previously published protocols described workflow ^47^. The CTF estimation for each tilt was performed by using emClarity version 1.4.3 , and the subvolumes were selected by using automatic template matching function within emClarity using reference derived from EMDB-21452 ^15^ that was low-pass filtered to 30-Å resolution in emClarity. The template matching results were cleaned manually by comparison of the binned tomograms overlaid with the emClarity-generated IMOD model showing the x,y,z coordinates of each cross-correlation peak detected. After manually template cleaning, a total of 450 subvolumes from the lamella dataset and a total of 7090 subvolumes from the extracellular viruses dataset were retained, deriving from 3 tilt-series and 50 tilt-series respectively, for the following averaging and alignment steps in emClarity.

For the extracellular viruses dataset, the 3D iterative averaging and alignment procedures were carried out gradually with binning of 4x, 3x, 2x , each with 2–3 iterations with increasingly restrictive search angles and translational shifts. Three-fold symmetry was applied during all the steps. Final converged average map was generated using bin2 tomograms with pixel size of 3.26 Å/pixel and a box size of 123×123×123 voxels. Resolution indicated by 0.143 FSC cut-off was 8.7 Å. The same process was carried out for lamella dataset, except for the final average map was generated with pixel size of 4.26 Å/pixel and a box size of 90×90×90 voxels and a final resolution at 11 Å (Gold standard FSC at 0.143 cut-off).

For the spikes inside transport vesicles, 55 subtomograms were iteratively averaged and aligned through 6 iterations (the first 4 with 4x binning, the last 2 with 3x binning). Three-fold symmetry was applied during all the steps. Final converged average map was generated using bin3 tomograms with pixel size of 6.39 Å/pixel and a box size of 72×72×72 voxels. Resolution indicated by 0.143 FSC cut-off was 24.4 Å.

### Serial cryoFIB/SEM Segmentation

Cell structures were manually segmented from stacks of images using ImageJ ^52^ and Microscopy Image Browser (MIB) software ^53^ on a Windows computer with 32GB RAM and Wacom Cintiq Pro display tablet with pen. Datasets of below 2GB in .mrc format were analysed one at a time, where one dataset comprised of 200 subsequent images on average.

### CryoET segmentation and 3D visualization

Transport vesicles, viral membrane, nuclear membrane, double membrane vesicles (DMV), and single membrane vesicles (SMV) were segmented using convolutional neural networks based tomogram annotation in the EMAN2.2 software package ^54^. Viral spikes were mapped back to their original particles position using emClarity tomoCPR function. UCSF Chimera ^55^ was used to visualize the segmentations and subtomogram average structures in 3D.

### Soft X-ray Cryo-tomography

Data were collected in areas of interest on vitrified samples on 3mm TEM grids according to established protocols ^38^. Grids were loaded on the X-ray microscope at B24 and were first mapped using visible light with a 20X objective. The resulting coordinate-map was used to locate areas of interest where 2D X-ray mosaics were collected (X-ray light used was at 500eV) and used to identify areas of interest within. Tilt series of 100–120º were collected for each field of view area of interest at 0.2 or 0.5º steps with constant exposure of 0.5 sec keeping average pixel intensity to between 5–30k counts. All tilt series were background subtracted, saved as raw tiff stacks and reconstructed using either IMOD ^50^ or Batchruntomo^56^.

### Quantification and statistical analyses

Number of portals in DMV and plasma membrane discontinuities were determined after visual inspection and manual counting by two independent investigators. The investigators were not blinded during experiments and outcome assessment.

## Supplementary Material

1

2

3

4

5

6

7

8

9

10

## Data Availability

The raw Serial cryoFIB/SEM images, soft X-ray cryo-tomograms, and cryoET tilt series of SARS-CoV-2 infected and uninfected cells will be deposited at EMPIAR (https://www.ebi.ac.uk/pdbe/emdb/empiar/). CryoEM density maps for prefusion SARS-CoV-2 Spike Protein from virions at assembly sites and those released from cells are deposited at the EMDB under accession code EMD-22976 and EMD-22977, respectively.
