# Supplementary material for: Correlative Multi-scale Cryo-imaging Unveils SARS-CoV-2 Assembly and Egress
Source: Res Sq. 2021 Jan 19:rs.3.rs-134794. Preprint. [Version 1] doi: 10.21203/rs.3.rs-134794/v1 (PMC7836121; doi:10.21203/rs.3.rs-134794/v1)
Supplement: 1 [file b8f74f7503a5d7d53305fd10.pdf]

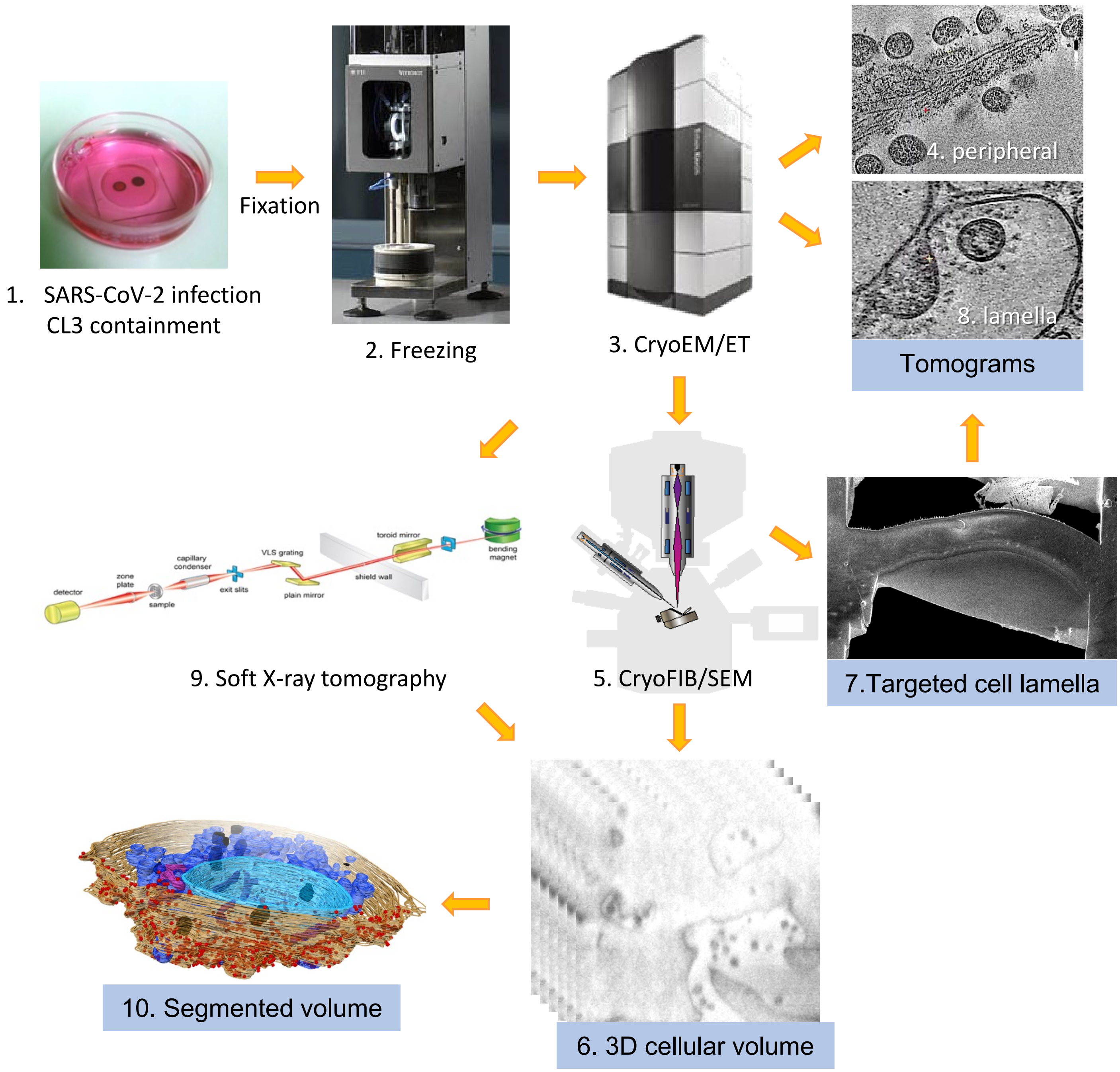

Figure S1 | A workflow for correlative multi-modal multi-scale imaging of SARS-CoV-2 infected cells. 1) Cells are grown on indexed EM grids, infected with SARS-CoV-2 and fixed with paraformaldehyde. 2) Grids are plunge-frozen in liquid ethane and 3) imaged by cryoEM/ET to locate the infected cells. 4) Tomograms are collected on the cell periphery of infected cells. 5) Infected cells are subjected to processing and imaging in a cryoFIB/SEM dualbeam instrument for 6) serial cryoFIB/SEM volume imaging and 7) targeted cell lamella. 8) Tomograms are collected from cell lamellae. 9) Alternatively, infected cells are imaged by soft-X-ray cryo-tomography. 10) Cellular volume data are manually segmented.

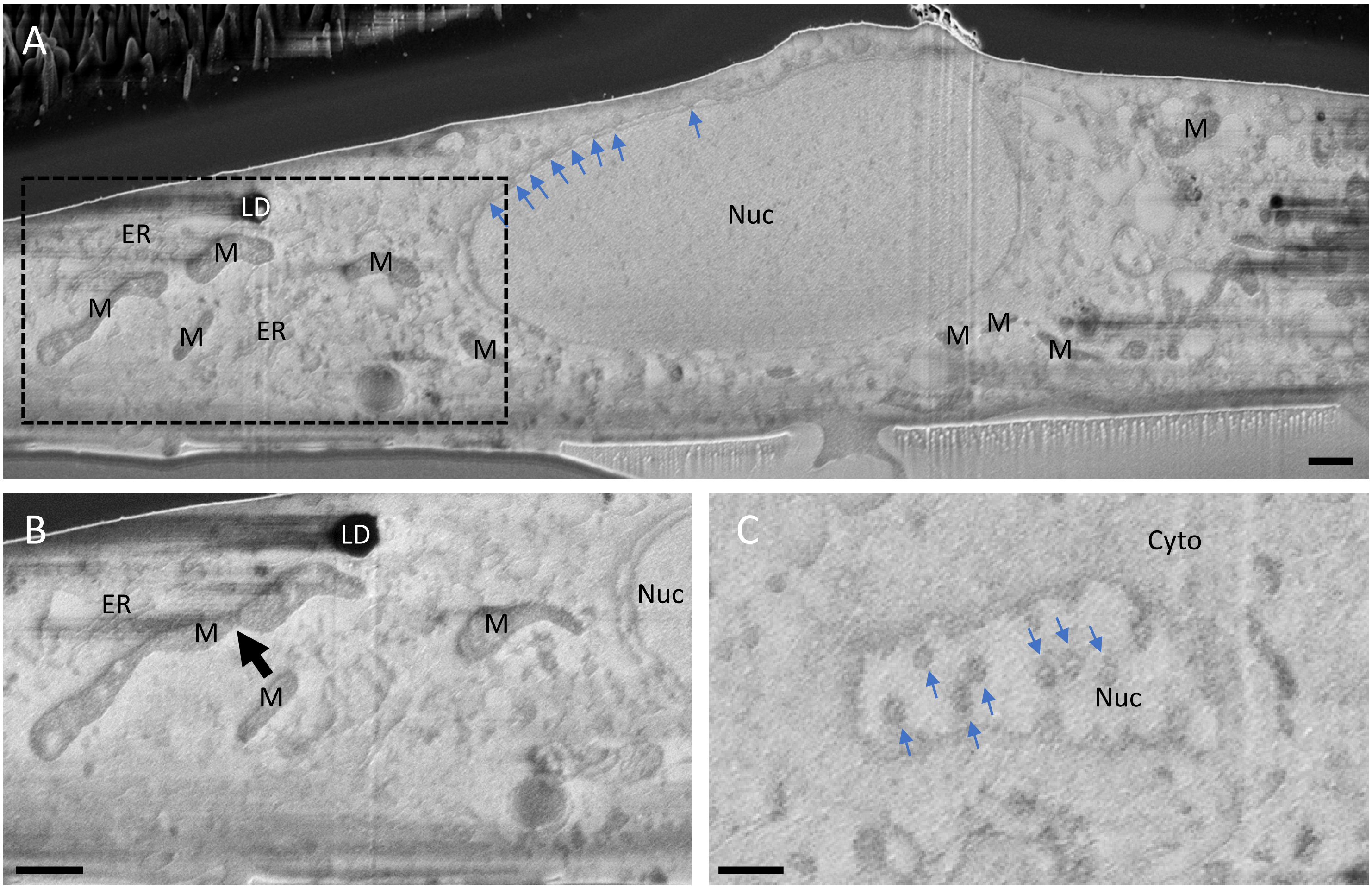

Figure S2 | Serial cryoFIB/SEM of control uninfected cell, related to Figure 1. (A) A representative overview of a cryoFIB/SEM slice from an uninfected cell. Blue arrows point to nuclear pores. (B) Detailed view of the dashed area in A from a slice 100 nm in depth depicting a connected mitochondrial network. (C) A cryoFIB/SEM slice tangential to the nuclear envelope, showing top view of nucleopores (blue arrows). Nuc, nucleus; Cyto, cytoplasm; ER, endoplasmic reticulum; M, mitochondria; LD, lipid droplet. Scale bars, 300 nm.

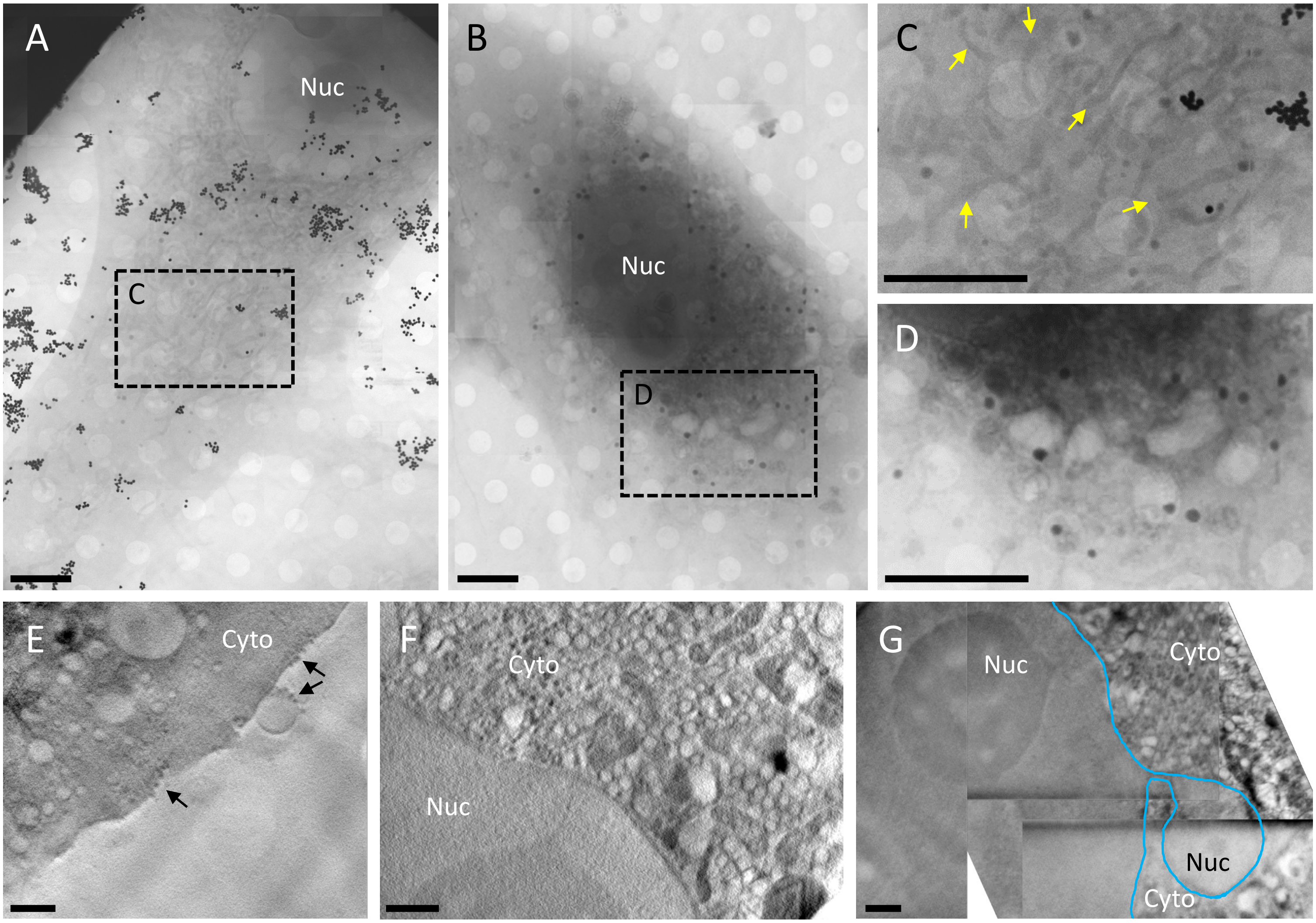

Figure S3 | Soft X-ray cryo-tomography of SARS-CoV-2 infected cells, related to Figure 1. (A-B) Soft X-ray overview mosaics of uninfected (A) and infected (B) Vero cells. (C-D) Detailed view of boxed area in A and B depicting mitochondrial network in uninfected (C) and fragmented mitochondria in infected cell (D) (yellow arrows). (E-F) Soft X-ray tomogram slices taken from infected cells depicting viruses at cell edge (E) (black arrows), abundant DMVs and a damaged mitochondria (F) (yellow arrow). (G) A montage of four tomograms depicting cytoplasmic invasion (or nuclear blebbing) in an infected cell. Nuclear envelope is outlined in cyan. Nuc, nucleus; Cyto, cytoplasm. Black dots in A and C are gold fiducial markers. Scale bar is 5 µm in A, B, C, D; 1 µm in E, F and G.

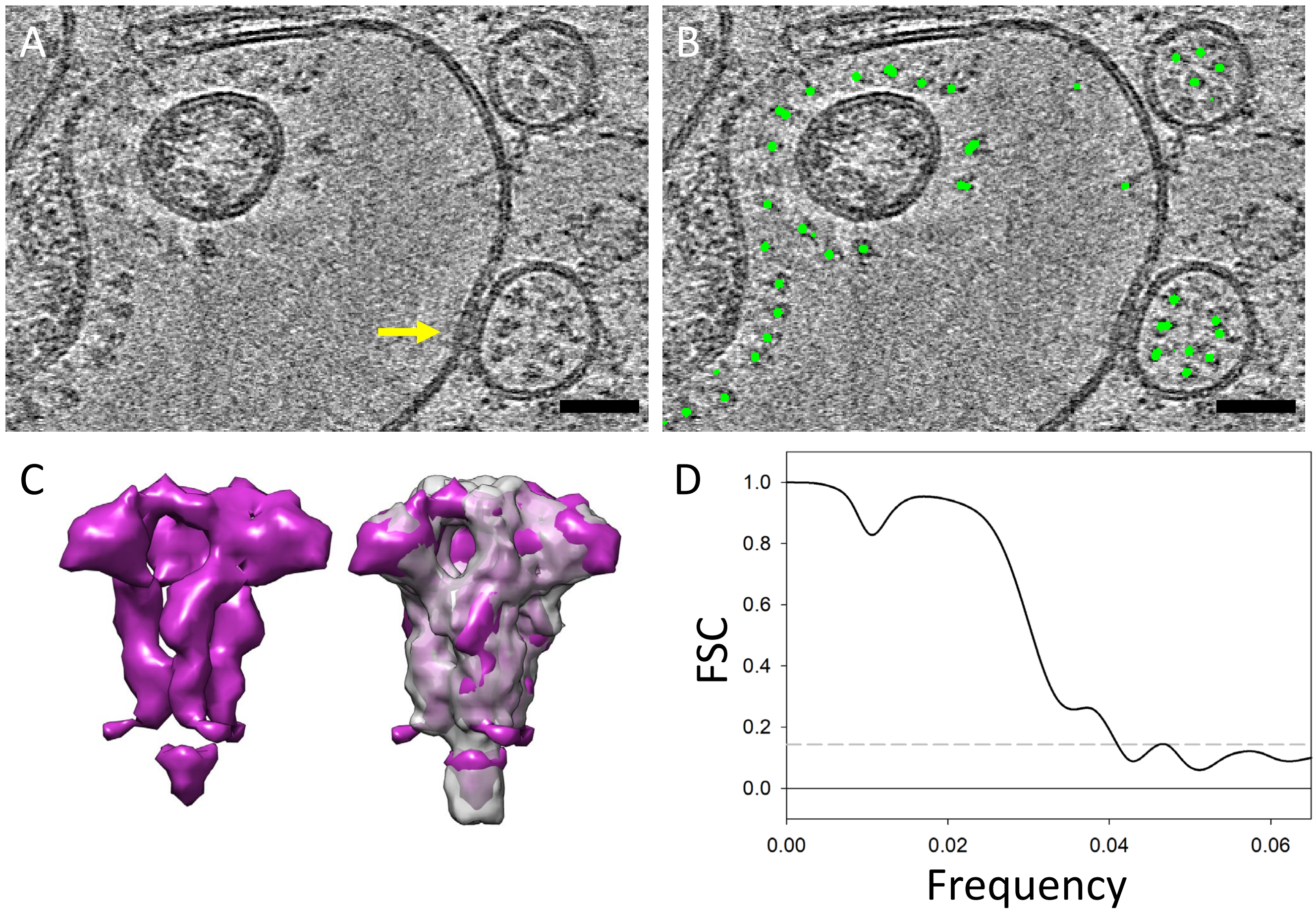

Figure S4 | Spike transporting vesicles. (A) A slice of tomogram from a cell lamella, depicting transporting vesicles next to an SMV. There appear electron densities connecting a transporting vesicle to the SMV (Yellow arrow). (B) Template matching of prefusion spike (green dots) overlaid with the tomogram slice in A. Spikes were found on the surface of virions, on the membrane of SMVs, and on the transporting vesicles. (C) Subtomogram average of spike from transporting vesicles (left) and overlapped with the density map of spike from virus particles released from cell (transparent gray) (right). A total of 55 subtomograms were aligned and averaged. (C) Fourier shell correlation (FSC) plot of subtomogram averaged spike density maps.

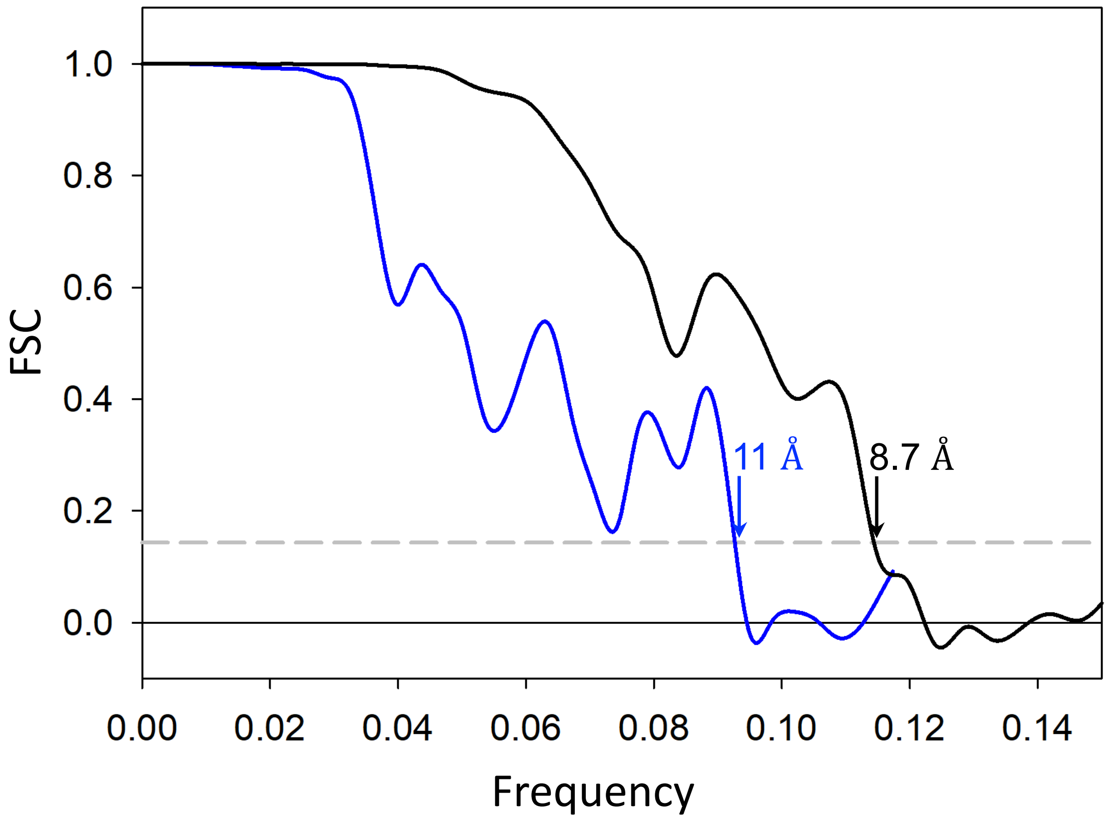

Figure S5 | Fourier shell correlation (FSC) plots, related to Figure 5. Fourier shell correlation (FSC) plots of subtomogram averaged spike density maps derived from intracellular virions (blue, from 450 subvolumes) and from released virions (black, from 7090 subvolumes). The dashed lines mark the FSC value of 0.143.
